# A Ministry of Public Health

**Published:** 1914-07-04

**Authors:** 


### A MINISTRY OF PUBLIC HEALTH.

In this country we are not exactly precipitous in the adoption of reforms, even when the case in favour of them is overwhelmingly strong. But in the end sane counsel and sober judgment are usually accepted and carried out, though weary years of educational effort have first to be undertaken. As an instance of what we mean, the creation of a Ministry of Public Health may be exemplified. Within the last few days two of the Cabinet—Lord Haldane and Mr. Masterman—have publicly referred to this office as one they hope to see in existence at no distant date. It can hardly be imagined that two such responsible exponents of official Liberalism would commit themselves to the expression of such a hope unless the matter had already been discussed. Nor is the question one of party politics: we feel certain that, were a change of Government to ensue, the advantages of a Ministry of Public Health would so speedily impress themselves upon His Majesty's new advisers that there would be little chance of any material deterioration of its chances.

To ourselves these signs of the times are of especial interest. For several years the establishment of such a Ministry, to unify and centralise the powers now wielded piecemeal and disconnectedly by three Government departments and hundreds of lesser authorities, has been persistently advocated in The Hospital. We need do no more than refer to the remarks on this subject which we made four years ago (The Hospital, July 16, 1910, p. 454), when we were obliged to admit that, in spite of long advocacy, "hitherto the creation of a special Health Department under Government has been a pious hope." Now the spade work of years, in which we are proud to have borne a part, however small, is apparently about to fructify; and if it does, those who believe that a Ministry of Public Health can be made an instrument of beneficent progress must see that every chance shall be given to it to achieve that end. Political and party considerations must be especially guarded against, both in the disposition of the powers and authority transferred from existing departments, and in the selection of the Parliamentary representation and the permanent staff of the Ministry. These problems of organisation are unquestionably difficult and formidable. It will be extremely hard, as we insisted four years ago, to assign to the future office a list of duties and responsibilities which will satisfy everyone, will not clash with those appertaining to other departments, and will tend to secure harmonious co-operation between the institutions, the medical profession, and the State. In particular, the voluntary hospitals will need to be hyper-jealous of the Ministry, for their future will be most vitally affected by the views which the Minister of Health for the time being may hold, and by the powers over them with which he may be entrusted. Enough has been said to show that this change, so desirable in itself, when it comes will need to be most carefully watched by all who have at heart the best interests of the public health.

